# Permeation of Biopolymers Across the Cell Membrane: A Computational Comparative Study on Polylactic Acid and Polyhydroxyalkanoate

**DOI:** 10.3389/fbioe.2020.00718

**Published:** 2020-06-30

**Authors:** Tommaso Casalini, Amanda Rosolen, Carolina Yumi Hosoda Henriques, Giuseppe Perale

**Affiliations:** ^1^Polymer Engineering Laboratory, Department of Innovative Technologies, Institute for Mechanical Engineering and Materials Technology, University of Applied Sciences and Arts of Southern Switzerland, Manno, Switzerland; ^2^Ludwig Boltzmann Institute for Experimental and Clinical Traumatology, Vienna, Austria

**Keywords:** molecular dynamics, lipid bilayer, permeation, molecular modeling, biopolymers

## Abstract

Polymeric nanoparticles, which by virtue of their size (1–1000 nm) are able to penetrate even into cells, are attracting increasing interest in the emerging field of nanomedicine, as devices for, e.g., drugs or vaccines delivery. Because of the involved dimensional scale in the nanoparticle/cell membrane interactions, modeling approaches at molecular level are the natural choice in order to understand the impact of nanoparticle formulation on cellular uptake mechanisms. In this work, the passive permeation across cell membrane of oligomers made of two employed polymers in the biomedical field [poly-D,L-lactic acid (PDLA) and poly(3-hydroxydecanoate) (P3HD)] is investigated at fundamental atomic scale through molecular dynamics simulations. The free energy profile related to membrane crossing is computed adopting umbrella sampling. Passive permeation is also investigated using a coarse-grained model with MARTINI force field, adopting well-tempered metadynamics. Simulation results showed that P3HD permeation is favored with respect to PDLA by virtue of its higher hydrophobicity. The free energy profiles obtained at full atomistic and coarse-grained scale are in good agreement each for P3HD, while only a qualitative agreement was obtained for PDLA. Results suggest that a reparameterization of non-bonded interactions of the adopted MARTINI beads for the oligomer is needed in order to obtain a better agreement with more accurate simulations at atomic scale.

## Introduction

The detailed knowledge of drug/membrane interactions plays a key role for the determination of the ADME (adsorption, distribution, metabolism and excretion) profile of active compounds. The efficacy of an administered drug also depends on its ability to cross cellular membranes or barriers of biological interest, such as the blood–brain barrier, to reach the desired target. Membrane permeation can occur through different mechanisms; passive diffusion (i.e., membrane crossing due to the concentration gradient) plays a key role when small uncharged molecules are involved (Smith et al., 2014) and its detailed understanding is essential for drug design. There are established experimental techniques and protocols for investigating drug permeation in model membranes, but their limited spatial resolution does not allow shedding light behind the specific interactions. Simulations at fundamental molecular level emerged as the ideal tool to improve our knowledge, thanks to the detail at atomic scale that allows highlighting the most relevant interactions behind the observed or expected permeation rate (Di Meo et al., 2016; Shinoda, 2016). A lipid bilayer is a heterogeneous environment because of the presence of polar head groups and hydrophobic chains (Nagle and Tristram-Nagle, 2000). These aspects can be accounted for, in detail, by means of simulations at molecular level, which allow developing mechanistic interpretations and models for lipophilic compounds permeation, as widely discussed by Dickson and coworkers (Dickson et al., 2017). In this regard, the growing use of computational techniques such as molecular dynamics (MD) simulations is the result of several aspects: First, the increasing availability of computational resources, coupled with software optimization, which lead to affordable and meaningful simulations. Second, the continuous development and improvement of accurate force fields tailored for lipid bilayers; indeed, the reliability of MD simulations outcomes is strongly dependent on the robustness of the chosen force field, whose importance cannot be underestimated. Third, it should be mentioned that membrane permeation usually involves an energy barrier much higher than the thermal energy *k_B_T* (where *k*_B_ is Boltzmann constant and *T* is absolute temperature) available to molecule in standard simulation at temperature *T*. This implies that a membrane crossing event would rarely be observed in a standard MD simulation, while multiple events should occur in a simulation in order to obtain statistically meaningful results. In other words, the characteristic time scale of molecule diffusion is larger than the time scale accessible to MD simulations. This issue can be overcome by means of enhanced sampling methods, which enhance the transition between metastable states separated by free energy barriers higher than *k_B_T*. The most popular method for drug/membrane interactions is umbrella sampling (US) (Torrie and Valleau, 1977), which allows obtaining the potential of mean force (PMF) as a function of a relevant reaction coordinate, usually taken as the distance between the center of the membrane and the center of mass of the molecule of interest. Position-dependent diffusion coefficients and permeation coefficients can be also obtained through the inhomogeneous solubility-diffusion model (ISDM) (Marrink and Berendsen, 1994). Such protocol is still widely employed nowadays for different systems of interest (Bochicchio et al., 2015; Dickson et al., 2017, 2019; Teixeira and Arantes, 2019). Another useful technique is constituted by well-tempered metadynamics (WTMD) (Barducci et al., 2008); briefly, WTMD allows recovering the free energy landscape of the system of interest as a function of few relevant degrees of freedom [commonly referred as collective variables (CV)] by adding a time-dependent bias. WTMD attracted some interest for the study of the permeation of small molecules, because of its increased computational efficiency with respect to US and to the possibility to add easily a bias potential to other CV that can play a role in membrane permeation, such as permanent orientation or intramolecular hydrogen bonds (Minozzi et al., 2011; Jambeck and Lyubartsev, 2013; Loverde, 2014; Saeedi et al., 2017). Simulations usually consider the interaction of a single drug molecule with a model membrane, usually made of dioleoylphosphatidylcholine (DOPC) or dipalmitoylphosphatidylcholine (DPPC), thanks to the availability of validated force fields (Dickson et al., 2014; Ingolfsson et al., 2016; Frederix et al., 2018). The use of a model membrane is an accepted approximation; adopting more realistic models still suffers from the lack of experimental data needed to validate force field parameters (Poger et al., 2016) but there is an increasing number of examples of heterogeneous membranes in literature. A common solution is the addition of cholesterol or other molecules in the model membrane (Murzyn et al., 2005; Hoopes et al., 2011; Tse et al., 2018). Recently, Tse et al. (2019) proposed a full atomistic model of a mammalian cell membrane, which contains 26 different components. The same considerations can be in principle extended also to biomaterials/membrane interactions, whose simulations are attracting an increasing interest because of the new paradigms introduced by nanomedicine. Indeed, simulations at fundamental molecular level, due to the involved time and length scales, are the natural modeling tool for improve our understanding of the interactions between nanocarriers (whose size is between 1 and 1000 nm) and biological components (proteins, carbohydrates, membranes, *et cetera*). Focusing on biomaterials/membrane interactions, on the one side, nanocarriers such as nanoparticles can cross the cellular membrane also through passive permeation. On the other side, when bioresorbable polymers are employed, degradation products can permeate through cellular membranes and accumulate into the cells, thus leading to adverse effects. Overall, this approach matches the requirements of the “safety by design” paradigm too. Because of the involved time and length scales, MD simulations with enhanced sampling methods are not always suitable to investigate nanoparticles/membrane interactions (Schulz et al., 2012; Casalini et al., 2019a) and coarse-grained (CG) models should be employed. As recently discussed (Ingolfsson et al., 2014; Lunnoo et al., 2019), CG models also allow including heterogeneous lipid bilayers, moving toward a more realistic description of the cellular membranes. Despite the loss of the atomic detail, they provide interesting insights if accurately parameterized against experimental data or full atomistic simulations (Marrink and Tieleman, 2013). Parameterization can be performed, e.g., by reproducing with a CG model the PMF of interested obtained with MD simulations (de Jong et al., 2013). In this work, we study by means of molecular dynamics simulations the diffusion across a DOPC model membrane of small oligomers made of poly-D,L-lactic acid (PDLA) and poly(3-hydroxydecanoate) (P3HD) chosen as representative compound of the family of polyhydroxyalkanoates (PHA). PDLA and PHA gained a wide interest in the biomedical field since they merge several interesting peculiarities, such as biocompatibility, good mechanical properties and an *in situ* degradation due to hydrolysis mechanism (Bassas-Galia et al., 2017; Butt et al., 2018; Casalini et al., 2019b). This led to the development of a wide range of biomedical devices, from bone fixation screws to nanoparticles for targeted drug delivery. On the one side, the excessive accumulation of degradation products inside cells may lead to adverse effects (Ramot et al., 2016); on the other side, a deeper understanding of the endocytic pathway for nanoparticle uptake can support the experimental design of new and more effective formulations. This constitutes the starting point of this work, which is structured as follows. First, the free energy landscape related to the permeation of small PDLA and P3HD oligomers (representative of degradation products from polymer hydrolysis) is obtained adopting umbrella sampling. Membrane crossing is subsequently simulated adopting a coarse-grained model and the free energy landscape is computed by means of WTMD. The assessment of the suitability of a coarse-grained model, parameterized on more accurate simulations at atomic scale, is fundamental to investigate the permeation of entire nanoparticles in model membranes, which would not be feasible with full atomistic simulations due to the involved time and length scales.

## Methods

### Force Field Parameterization

The second-generation general amber force field (GAFF2) (Wang et al., 2004) was employed for PDLA and P3HD. Atomic charges were computed by means of restrained electrostatic potential (RESP) method (Bayly et al., 1993; Cornell et al., 1993), consistently with force field parameterization procedure. Oligomers composed of 6 monomer units were optimized *in vacuo* through density functional theory (DFT) calculations at B3LYP/6-31G(d,p) level of theory. The obtained conformations were subsequently employed to compute electrostatic potentials *in vacuo* at HF/6-31G^∗^ level of theory. Calculations were performed my means of Gaussian09 software (Frisch et al., 2016). Atomic charges were then fitted by means of RESP procedure, adopting a two-step protocol. First, partial atomic charges were calculated starting from the previously obtain electrostatic potential values, imposing an overall charge value equal to zero. In the second step, charge equivalence is imposed for chemically equivalent atoms. This procedure allowed obtaining a library of building blocks that can be used to build polymer chains of different length. Lipid17 force field (Dickson et al., 2014) was adopted for DOPC lipid bilayer because of its validated parameters. TIP3P water model (Jorgensen et al., 1983) was employed for explicit solvent molecules, consistently with force field parameterization. Parameters for monovalent ions, optimized for TIP3P model, were taken from Joung and Cheatham (2008, 2009). Details are reported in Supplementary Material.

### Creation of the Molecular Models

Polymer chains were built using *tLeap* module included in *AmberTools*; chain ends were saturated with methyl groups. The same tool was used to solvate with TIP3P water molecules and add ions to assure electroneutrality, where needed. A DOPC lipid bilayer composed of 128 DOPC molecules was assembled and solvated by means of CHARMM-GUI web server (Wu et al., 2014; Lee et al., 2019). The membrane lies on *xy*-plane and water molecules were placed only along *z* direction, so that an infinite surface can be obtained by applying periodic boundary conditions. A suitable number of Na^+^ and Cl^–^ was added to reach 0.15 M salt concentration, which mimics physiological conditions. The bilayer/polymer system was assembled starting from equilibrated configurations of the single components by means of *AddToBox* module included in *AmberTools*; ions were added in order to mimic physiological conditions. A coarse-grained model was built adopting MARTINI force field (Marrink et al., 2007), chosen for its validated results and its straightforward parameterization procedure. A bilayer composed of 128 DOPC molecules was built by means of CHARMM-GUI web server, similarly to the full atomistic model, with explicit water and ions beads. Parameters for bonded and non-bonded interactions of DOPC molecules are already available in MARTINI library. PDLA was coarse-grained by adopting 7 C5 beads, while P3HD was modeled using 7 Na beads for the backbone and one C1 bead and one C3 bead for each side chain. Structures are depicted in Figures 4A,B.

Parameters for bonded interactions were computed to best reproduce the bond, angle dihedral distributions obtained with MD simulations at full atomistic level. Parameters for non-bonded interactions were taken from MARTINI library. Details are reported in Supplementary Material.

### Molecular Dynamics Simulations

Molecular dynamics simulations were performed according to the following protocol. First, energy minimization step procedure was carried out by fixing the solute with a harmonic restraint (force constant equal to 500 kcal mol^–1^ Å^–2^), in order to remove bad solvent/solvent and solute/solvent contacts due to the random placement of water molecules. Energy minimization was subsequently repeated removing the restraint on solute molecules. Temperature was raised from 0 to 310 K by means of 20 ps in NVT ensemble (constant number of particles *N*, volume *V*, and temperature *T*). When the lipid bilayer was present in the simulation box, temperature was slowly increased from 0 to 310 K through 10 ns in NVT ensemble adopting a linear ramp. Solute was kept fixed through a weak harmonic restraint (force constant equal to 10 kcal mol^–1^ Å^–2^); temperature was maintained to the desired production value by means of Langevin thermostat, adopting a collision frequency equal to 1 ps^–1^. Finally, system equilibration was achieved by means of molecular dynamics simulations in NPT ensemble (i.e., at constant number of particles *N*, pressure *P*, and temperature *T*) at 310 K and 1 atm. Pressure was controlled by means of isotropic (for polymer/water systems) and anisotropic (for systems containing lipid bilayer) Berendsen barostat. Simulations were performed adopting periodic boundary conditions; the chosen cutoff value for long-range interactions was set equal to 1 nm. Particle Mesh Ewald (PME) was chosen for treating electrostatic interactions. SHAKE algorithm was employed to constrain all covalent bonds involving hydrogen atoms; this allowed propagating system dynamics through Leap-Frog algorithm using a time step equal to 2 fs. Simulations were carried out with GPU cards using the *pmemd.cuda* module implemented in AMBER 16 (Salomon-Ferrer et al., 2013; Case et al., 2016). A summary of performed MD simulations is reported in Supplementary Material.

### Umbrella Sampling

Umbrella sampling was performed by choosing the distance between the center of mass (COM) of oligomer chain and the center of the lipid bilayer as the relevant reaction coordinate (Di Meo et al., 2016; Shinoda, 2016). Simulations were carried out using 41 windows, covering a distance range from 0 to 40 Å with a spacing value equal to 1 Å.

Oligomers were restrained to the reference distance of each window by means of a harmonic potential with a force constant equal to 2.5 kcal mol^–1^ Å^–2^, chosen so that a good overlap between distance distributions among adjacent windows could be obtained. Only the *z* component of the distance was subjected to the restraint, while oligomers were free to move along *x* and *y* directions.

First, the oligomer was placed in the center of the membrane by applying a harmonic potential and 40 ns MD simulation in NPT ensemble were carried out to reach equilibration. Then, Umbrella Sampling simulations were performed so that the oligomer was pulled out from membrane center to the external environment; indeed, it has been shown in scientific literature that this procedure (rather than gradually placing a molecule in the bilayer) improves the convergence of the results (Filipe et al., 2014); 80 ns MD simulations in NPT ensemble at 1 atm and 310 K were carried out for each window, leading to 3.2 μs of total sampling time for each system.

Free energy as a function of the chosen reaction coordinate was obtained by means of weighted histogram analysis method (WHAM) (Kumar et al., 1992; Roux, 1995), using a 0–40 Å distance grid with a grid spacing equal to 0.025 Å; a further decrease of grid spacing did not lead to appreciable variations of the obtained results.

For each window, the first 50 ns were used for system equilibration and discarded; free energy was computed using the last 30 ns, using three blocks of 10 ns each. Results are expressed as average ± standard deviation. Details are reported in Supplementary Material.

A position-dependent diffusion coefficient can be computed by means of inhomogeneous solubility-diffusion model (Marrink and Berendsen, 1994), which was applied in literature to small solutes (water, methanol, etc.) (Bemporad et al., 2004; Orsi et al., 2009; Lee et al., 2016) as well as to drug-like molecules (Dickson et al., 2017). Diffusivity as a function of z coordinate *D(z)* can be computed as follows (Hummer, 2005):

(1) $$D\left(z\right)=\frac{var{\left(z\right)}^{2}}{\int_{0}^{\infty }C_{\text{zz}}\left(t\right)dt}$$

where *var*(*z*) is the variance of the *z*-component of the distance in a US window and *C*_zz_ is the position autocorrelation function, defined as follows:

(2) $$C_{\text{zz}}\left(t\right)=\delta z\left(0\right)\delta z\left(t\right)$$

(3) δz(t)=z(t)-z

where <*z*> is the average value of the distance in the US window.

Autocorrelation function was numerically integrated by means of *trapz* algorithm implemented in MATLAB (which takes advantage of the trapezoidal rule) until it decayed to var(*z*)⋅10^–2^, in order to exclude from the integration the noise around *C*_zz_(*t*) = 0 (Dickson et al., 2017). The position-dependent resistance can be also computed:

(4) $$R\left(z\right)=\frac{\text{exp}\left(\beta \Delta G\left(z\right)\right)}{D\left(z\right)}$$

where β is equal to (*k_B_T*)^–1^, *k*_B_ is Boltzmann constant, *T* is the absolute temperature, and Δ*G*(*z*) is the free energy profile. While *D*(*z*) is evaluated for every window (41 values), Δ*G*(*z*) is obtained for every grid point. Therefore, in order to compute *R*(*z*), the diffusion coefficient is evaluated along the distance grid using a shape-preserving interpolant by means of *mpich* algorithm implemented in MATLAB. An overall permeation coefficient can be obtained by integrating the resistance profile:

(5) $$P_{eff}=\frac{1}{R_{eff}}=\frac{1}{\int_{-z_{\text{B}}}^{z_{\text{B}}}R\left(z\right)dz}$$

where the integration boundaries are referred to water phase at either side of the lipid bilayer (i.e., *z*_B_ = 40 Å and *–z_B_* = -40 Å). Binding free energy Δ*G*^0^_Bind_ and membrane partitioning constant *K*_*lip*_ can be also obtained:

(6) $$\Delta G_{\text{Bind}}^{0}=-k_{\text{B}}Tln\left(\frac{1}{z_{\text{B}}}\int_{0}^{z_{\text{B}}}\exp\left(-\beta \Delta G\left(z\right)\right)dz\right)$$

(7) $$K_{lip}=\text{exp}\left(-\beta \Delta G_{\text{Bind}}^{0}\right)$$

### Coarse-Grained Simulations

Simulations were performed with GROMACS 2018.3 (Pall et al., 2015). Focusing on DOPC bilayer, after energy minimization the system was progressively equilibrated by running five simulations of 10 ns each in NPT ensemble at 310 K and 1 atm. A harmonic restraint was applied to lipid molecules, reducing the value of the force constant in each simulation; force constant values equal to 200, 100, 50, 20 and 10 kJ nm^–2^ mol^–1^ were chosen for this purpose. Temperature and pressure were controlled by means of velocity rescaling algorithm (Bussi et al., 2007) and semiisotropic Berendsen barostat, respectively, with coupling time constants equal to 1 and 12 ps. Finally, 600 ns in NPT ensemble at 310 K and 1 atm was performed, adopting velocity rescaling algorithm and semiisotropic Parrinello-Rahman barostat (Parrinello and Rahman, 1981) for temperature and pressure control, respectively. Coupling time constants were not modified. A cutoff value equal to 1.1 nm was chosen for long-range electrostatic and Van der Waals interactions, which were computed adopting a reaction field (with a dielectric constant beyond the cutoff equal to 15) and a straight cutoff. A potential modifier was applied to VdW interactions to better perform with the Verlet cutoff scheme. Periodic boundary conditions were applied along *x*, *y*, and *z* directions; dynamics were propagated using Leap-Frog algorithm using a time step equal to 20 fs.

WTMD simulations were carried out with GROMACS 2018.3 patched with PLUMED 2.5 (Tribello et al., 2014). PDLA and P3HD were added in the water phase in the simulation box of the equilibrated DOPC bilayer, replacing water beads if necessary, with the *insert-molecule* tool implemented in GROMACS. After energy minimization and a brief equilibration (20 ns) in NPT ensemble at 310 K and 1 atm, WTMD simulations were carried out, considering the component along *z* direction of the distance between oligomer and bilayer centers of mass. The initial Gaussian height, sigma, and bias factor values were set equal to 0.8 kJ mol^–1^, 0.05 Å, and 30, respectively. Bias potential was added every 5000 steps (100 ps). Harmonic potentials were applied by means of *upper_walls* and *lower_walls* algorithms implemented in PLUMED (force constant equal to 50 kJ mol^–1^ nm^–2^) in order to promote membrane crossing events and to limit the CV exploration in a range of values of interest, i.e., between -45 and 45 Å.

The convergence of the free energy landscape was evaluated with two different methods, that is, checking the free energy difference as a function of simulation time and computing the error according to Bonomi et al. (2009):

(8) $$\varepsilon^{2}\left(t\right)=\frac{1}{vol\left(\text{Ω}\right)}\int ds{\left[V\left(s,t\right)-F\left(s\right)\right]}^{2}$$

where ε is the error, *t* is time, *s* represents the chosen collective variables, Ω is the explored CV region, *V*(*s,t*) is the external bias added to the system, and *F*(*s*) is the reference free energy profile, i.e., the one obtained at the end of the simulation. Plots are reported in Supplementary Material.

Free energy profiles as well as binding free energies were computed as an average of the last 2000 ns.

## Results and Discussion

### Oligomers and Lipid Bilayer Equilibration

**FIGURE 1:**
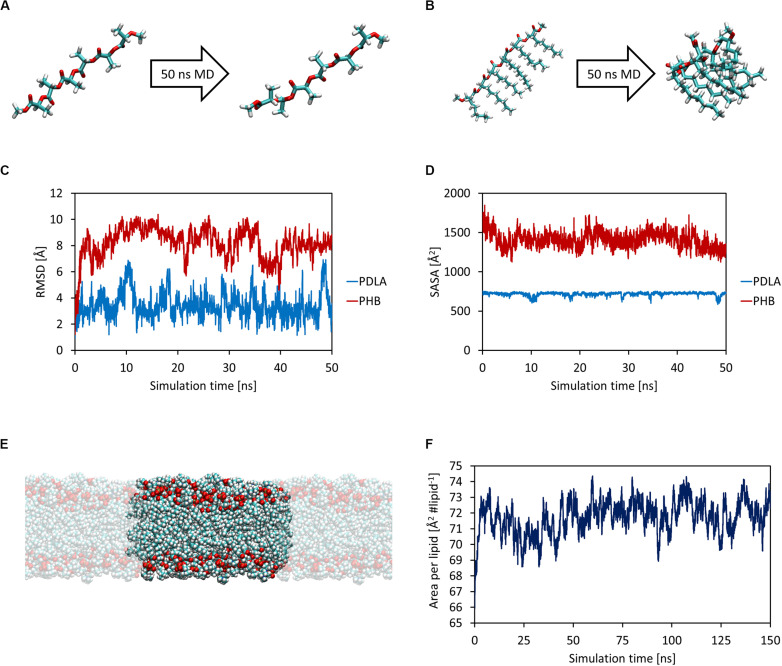
Equilibrated structures of PDLA **(A)** and P3HD **(B)** oligomers. RMSD **(C)** and SASA **(D)** as a function of simulation time. Equilibrated bilayer structure along with its periodic images that result in an infinite surface that lies on xy-plane **(E)**. Area per lipid as a function of simulation time **(F)**.

First, MD simulations were carried out in order to obtain equilibrated structures of both oligomers and the DOPC lipid bilayer, which mimics a cellular membrane. Each oligomer was equilibrated with 50 ns MD simulations in NPT ensemble at 1 atm and 310 K (Figures 1A,B). The attainment of reasonable equilibrated structures was checked by computing the root mean square displacement (RMSD) and the solvent accessible surface area (SASA) as a function of simulation time, as shown in Figures 1C,D. While PDLA oligomer did not experience substantial structural variations, P3HD oligomer folded due to its increased hydrophobicity related to aliphatic side chains. Focusing on DOPC bilayer, 150 ns MD simulations were performed for equilibration and the attainment of an equilibrated structure (Figure 1E) was verified by computing the area per lipid (Figure 1F) and membrane thickness (computed from the peak-to-peak distance of electron density profiles) as a function of simulation time. Equilibration led to an area per lipid and membrane thickness values equal to 72.04 ± 0.88 Å^2^ lipid^–1^ and 35.75 ± 0.45 Å, respectively; values are expressed as average ± standard deviation. The obtained values are consistent with the reported computational and experimental data provided by Dickson et al. (2014).

The equilibrated structures were thus employed for the study of oligomers permeation in the lipid bilayer.

### Oligomers Permeation

**FIGURE 2:**
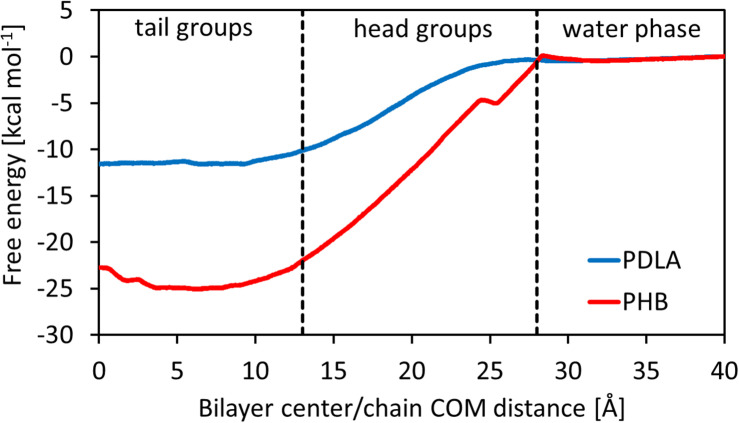
Free energy as a function of the distance between oligomer COM and bilayer center.

The main outcome from Umbrella Simulations is the free energy landscape as a function of the *z*-component of the distance between the center of mass of the oligomer and the center of the membrane. In this regard, it is possible to identify three different zones, related to the heterogeneous environment of the bilayer: tail groups (0 < *z* < 13 Å), head groups (13 < *z* < 27 Å) and water phase (27 < *z* < 40 Å). Results are shown in Figure 2. PDLA and P3HD free energy landscapes are consistent with the results shown in literature for hydrophobic molecules (Bemporad et al., 2004; Orsi et al., 2009; Bochicchio et al., 2015), since such oligomers preferably partition inside the membrane. Indeed, Δ*G*^0^_Bind_ computed through equation 6 is equal to -11.49 ± 0.69 and -23.85 ± 0.99 kcal mol^–1^ for PDLA and P3HD, respectively. The more favorable value related to P3HD is due to the relevant interactions between polymer/bilayer aliphatic chains. The minimum of the free energy lies in the region with the tail groups, by virtue of hydrophobic effects. Free energy increases moving toward the hydrophilic head groups, where no favorable interactions take place since no hydrogen bonds can be formed.

**FIGURE 3:**
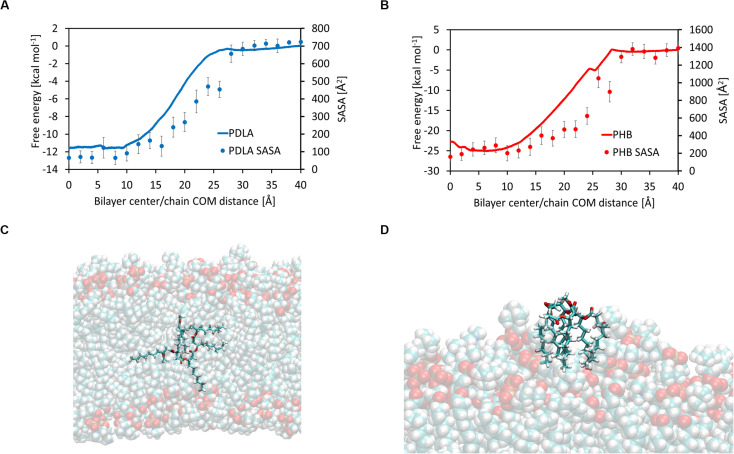
Free energy and solvent accessible surface area for PDLA **(A)** and P3HD **(B)**. Unfolded P3HD oligomer inside lipid bilayer **(C)** and at bilayer/water interface **(D)**.

Hydrophobic effects behind the free energy landscape can be highlighted through SASA values, computed using the last 10 ns of each window, as shown in Figures 3A,B; indeed, free energy profiles for PDLA and P3HD exhibit the same trend of SASA decrease due to permeation. Notably, P3HD oligomer also experiences unfolding inside the bilayer (Figure 3C), when it is surrounded by the hydrophobic tails. In addition, P3HD is still unfolded at the bilayer/water interface (Figure 3D); aliphatic chains point toward the lipid bilayer, while the backbone is exposed to the solvent. Up to authors’ best knowledge, experimental diffusion coefficients are not available, while computational studies are usually focused on smaller molecules. Comparison with literature data reveals that P3HD and PDLA diffusion coefficients are about two orders of magnitude lower if compared to low molecular weight compounds (ranging from water to benzene) or small drugs (Orsi et al., 2009; Dickson et al., 2017) and can be considered acceptable. The resistance as a function of collective coordinate reaches it minimum value in the center of the bilayer (by virtue of the favorable interactions) and it is maximum at water/bilayer interface. Indeed, polar head groups are the major obstacle to permeation, due to the not favorable interactions with the oligomers. All computed values are reported in Supplementary Material.

### Coarse-Grained Simulations

**FIGURE 4:**
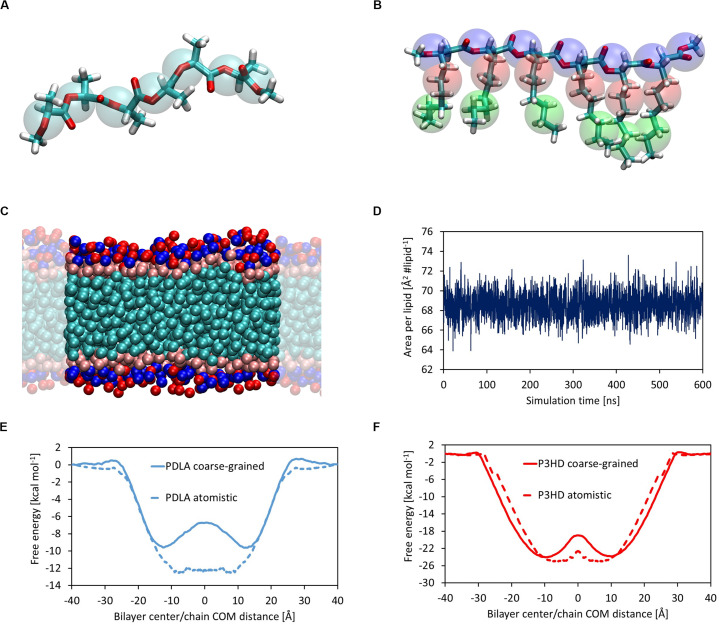
Coarse-grained representation of PDLA oligomer; C5 MARTINI beads are represented as transparent cyan spheres **(A)**. Coarse-grained representation of P3HD oligomer; Na, C3, and C1 MARTINI beads are represented as transparent blue, red, and green spheres, respectively **(B)**. Equilibrated structure of DOPC CG model **(C)**. Area per lipid as a function of simulation time for DOPC bilayer CG model **(D)**. Comparison of free energy profiles obtained from full atomistic and coarse-grained simulations for PDLA **(E)** and P3HD **(F)**. Profile from full atomistic simulations was mirrored for the sake of clarity.

The first step was evaluating the attainment of an equilibrated bilayer structure at CG level (Figure 4C) and its agreement with the outcomes from atomistic simulations. The average values of area per lipid and membrane thickness are equal to 68.57 ± 1.25 Å^2^ lipid^–1^ and 36.67 ± 0.57 Å, respectively, in good agreement with the results obtained at full atomistic level. It should also point out that in this case membrane, thickness was computed from the distance between the beads representative of the phosphate groups. Moreover, an equilibrated structure is rapidly obtained, as shown by the time evolution of the area per lipid (Figure 4D).

Free energy landscapes were obtained by means of WTMD; thanks to the higher accessible time scales provided by the intrinsic computational efficiency of CG simulations with respect to full atomistic ones, the sampling was performed considering the full CV range from -40 to 40 Å, in order to observe multiple membrane crossing events. The comparison between free energy profiles from full atomistic and coarse-grained simulations is shown in Figures 4E,F for PDLA and P3HB, respectively.

While the agreement for P3HD is good from both a qualitative and a quantitative point of view, only a fair qualitative agreement was obtained for PDLA. This is evident also focusing on Δ*G*_Bind_ values, which were computed also from CG simulations using the last 2000 ns. The value obtained for P3HD, equal to -22.69 ± 0.23 kcal mol^–1^ is in good agreement with the estimation from US, equal to -23.85 ± 0.99 kcal mol^–1^. On the other hand, the analogous comparison for PDLA oligomer showed an expected poor agreement, by virtue of Δ*G*_Bind_ values equal to -8.33 ± 0.11 and -11.49 ± 0.69 kcal mol^–1^ obtained from coarse-grained and full atomistic simulations, respectively.

The observed disagreement for PDLA results can be explained by taking into account the parameterization of non-bonded interactions of MARTINI beads. Indeed, PDLA has a backbone composed of ester bonds, which act as polar groups, and hydrophobic side chains constituted by methyl groups. The parameterization of the non-bonded interactions of the chosen MARTINI bead is thus not able to account for this balance, since the hydrophobicity in underestimated. Modeling PDLA polymer with Na MARTINI beads, representative of ester bonds only, leads to physically not consistent results: preliminary explorative simulations showed that the oligomer would preferably partition in water phase. On the other hand, more hydrophobic beads essentially take into account aliphatic backbones and would provide an overestimation of the affinity for the lipid phase.

Summarizing, while C5 MARTINI beads for PDLA polymer represent the best compromise, they do not provide a description of the polymer at CG level with an acceptable accuracy level. Therefore, while the CG model for P3HD presented here could be readily used to simulate an entire nanoparticle, a reparameterization of non-bonded interactions for PDLA oligomer is needed to improve the agreement with more accurate atomistic simulations.

## Conclusion

In this study, the passive permeation of small oligomers of polymer of interest in the biomedical field was studied by means of molecular dynamics simulations, at both full atomistic and coarse-grained level.

Simulations at atomic scale allowed obtaining the free energy landscape as a function of the distance between the center of the membrane and the center of mass of PDLA and P3HD, chosen as collective coordinate. Results showed that both oligomers preferably partition into the membrane; this trend could be explained in terms of hydrophobic effects by computing the solvent accessible surface area as a function of the collective coordinate.

The obtained free energy landscape can be in principle employed to tune a coarse-grained model, which can be used to simulate the permeation of an entire nanoparticle into a lipid bilayer, by virtue of the higher accessible time and length scales. For this reason, coarse-grained simulations were performed using MARTINI force field, to check whether the free energy landscape from atomistic simulations could be reproduced without further reparameterization.

Results showed a good quantitative agreement for P3HD oligomer and only a fair qualitative agreement for PDLA, highlighting the need of a further reparameterization of non-bonded interactions in order to better account for the hydrophobicity due to the methyl side groups.

## Data Availability Statement

All datasets generated for this study are included in the article/Supplementary Material.

## Author Contributions

TC, AR, and CH performed simulations and post processing. TC wrote the first draft of the manuscript. GP contributed to supervision of the work. All authors discussed and approved the contents of the manuscript and contributed to its final version by reading and editing.

## Conflict of Interest

The authors declare that the research was conducted in the absence of any commercial or financial relationships that could be construed as a potential conflict of interest.
