# Supplementary material for: Permeation of Biopolymers Across the Cell Membrane: A Computational Comparative Study on Polylactic Acid and Polyhydroxyalkanoate
Source: Front Bioeng Biotechnol. 2020 Jun 30;8:718. doi: 10.3389/fbioe.2020.00718 (PMC7344160; doi:10.3389/fbioe.2020.00718)
Supplement: Supplementary file 1 [file Data_Sheet_1.docx]

Supporting information for:

Biopolymers permeation across cellular membrane: a computational comparative study on polylactic acid and polyhydroxybutyrate

Tommaso Casalini^1*^, Amanda Rosolen^1^, Carolina Yumi Hosoda Henriques^1^, Giuseppe Perale^1,2^

^1^Polymer Engineering Laboratory, Institute for Mechanical Engineering and Materials Technology, Department of Innovative Technologies, University of Applied Sciences of Southern Switzerland, Manno, Switzerland

^2^Ludwig Boltzmann Institute for Experimental and Clinical Traumatology, Vienna, Austria

*** Correspondence:**Dr. Tommaso Casalini
tommaso.casalini@supsi.ch

*S.1 Atomic charges for poly(3-hydroxydecanoate)*

Charge derivation procedure led to the creation of three fragments, called 3PI, 3PH and 3PF for the first residue of the chain, bulk monomers and terminal residue of the chain, respectively (Figure S.1).


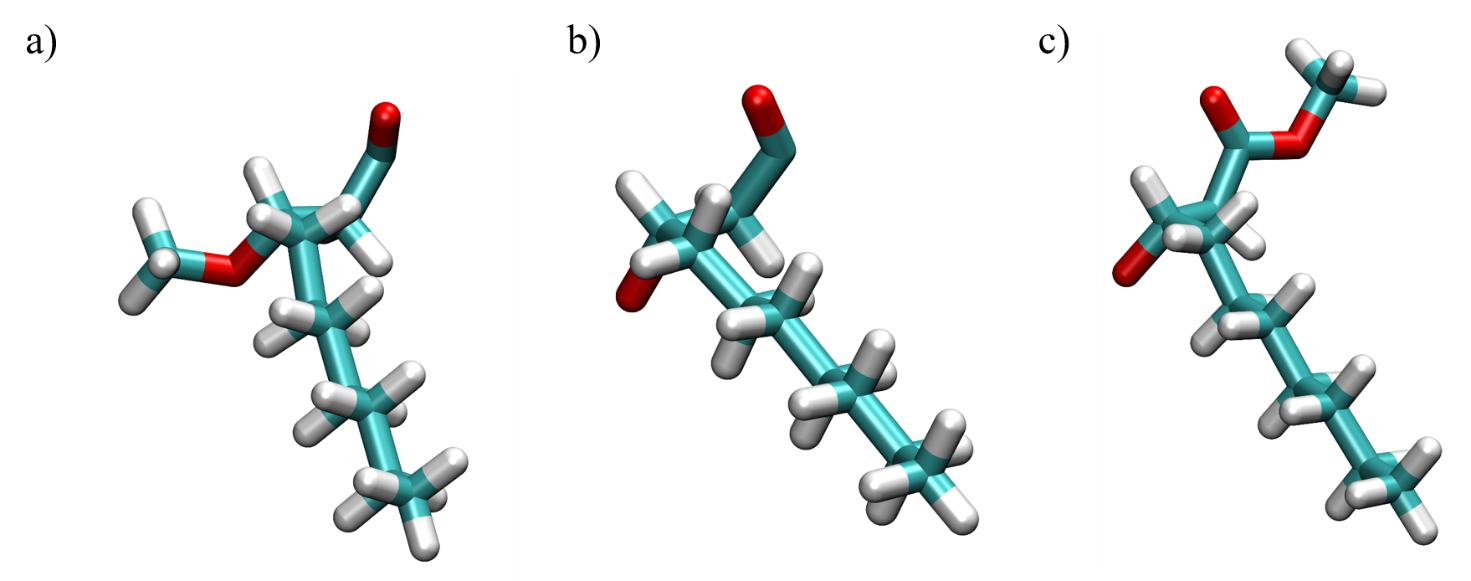


***Figure S.1.*** Building blocks for PHB: 3PI (a), 3PH (b) and 3PF (c).

Atom names, atom types (according to second generation GAFF force field), atomic charges and coordinates are reported in Table S.1 – S.3.

***Table S.1.*** Atom names, atom types, coordinates and charges for 3PI residue.

| **Atom name** | **Atom type** | **X** | **Y** | **Z** | **Charge** |
| --- | --- | --- | --- | --- | --- |
| C1 | c3 | -6.469 | -4.954 | 1.559 | -0.024699 |
| H1 | h1 | -6.787 | -5.292 | 2.549 | 0.064177 |
| H2 | h1 | -6.144 | -5.828 | 0.974 | 0.064177 |
| H3 | h1 | -7.333 | -4.503 | 1.052 | 0.064177 |
| O1 | os | -5.41 | -4.039 | 1.759 | -0.376592 |
| C2 | c3 | -4.761 | -3.611 | 0.558 | 0.195931 |
| C3 | c3 | -3.43 | -3.014 | 1.025 | 0.02251 |
| H4 | h1 | -4.535 | -4.491 | -0.065 | 0.043429 |
| C4 | c3 | -5.63 | -2.653 | -0.275 | -0.044859 |
| C5 | c | -2.44 | -2.77 | -0.094 | 0.44417 |
| H5 | hc | -3.58 | -2.081 | 1.578 | -0.003262 |
| H6 | hc | -2.969 | -3.707 | 1.738 | -0.003262 |
| H7 | hc | -5.051 | -2.361 | -1.158 | 0.003417 |
| H8 | hc | -6.492 | -3.215 | -0.659 | 0.003417 |
| C6 | c3 | -6.139 | -1.415 | 0.475 | 0.080808 |
| C7 | c3 | -7.103 | -0.567 | -0.365 | 0.022235 |
| H9 | hc | -6.635 | -1.733 | 1.4 | -0.009096 |
| H10 | hc | -5.294 | -0.786 | 0.785 | -0.009096 |
| C8 | c3 | -7.628 | 0.674 | 0.367 | -0.001563 |
| H11 | hc | -6.602 | -0.257 | -1.293 | -0.008687 |
| H12 | hc | -7.954 | -1.19 | -0.678 | -0.008687 |
| C9 | c3 | -8.597 | 1.515 | -0.472 | 0.023539 |
| H13 | hc | -8.128 | 0.362 | 1.295 | -0.010659 |
| H14 | hc | -6.779 | 1.299 | 0.677 | -0.010659 |
| C10 | c3 | -9.126 | 2.756 | 0.258 | 0.051141 |
| H15 | hc | -8.097 | 1.828 | -1.4 | -0.0074 |
| H16 | hc | -9.446 | 0.89 | -0.784 | -0.0074 |
| C11 | c3 | -10.093 | 3.591 | -0.588 | -0.072016 |
| H17 | hc | -9.627 | 2.444 | 1.185 | -0.008307 |
| H18 | hc | -8.278 | 3.382 | 0.569 | -0.008307 |
| H19 | hc | -10.45 | 4.468 | -0.04 | 0.012151 |
| H20 | hc | -9.611 | 3.945 | -1.506 | 0.012151 |
| H21 | hc | -10.97 | 3.003 | -0.883 | 0.012151 |
| O3 | o | -2.592 | -3.09 | -1.255 | -0.50503 |

***Table S.2.*** Atom names, atom types, coordinates and charges for 3PH residue.

| **Atom name** | **Atom type** | **X** | **Y** | **Z** | **Charge** |
| --- | --- | --- | --- | --- | --- |
| O2 | os | -1.331 | -2.156 | 0.382 | -0.47027 |
| C12 | c3 | -0.264 | -1.869 | -0.571 | 0.033396 |
| C13 | c3 | 1.028 | -2.014 | 0.234 | -0.080478 |
| H22 | h1 | -0.296 | -2.63 | -1.352 | 0.081515 |
| C14 | c3 | -0.497 | -0.498 | -1.216 | -0.036101 |
| H23 | hc | 0.299 | -0.349 | -1.955 | 0.034207 |
| H24 | hc | -1.434 | -0.563 | -1.781 | 0.034207 |
| C15 | c3 | -0.559 | 0.693 | -0.25 | 0.035173 |
| C16 | c3 | -0.903 | 2.009 | -0.96 | -0.001332 |
| H25 | hc | -1.304 | 0.493 | 0.53 | 0.006125 |
| H26 | hc | 0.403 | 0.813 | 0.268 | 0.006125 |
| C17 | c3 | -0.954 | 3.217 | -0.018 | 0.003849 |
| H27 | hc | -0.164 | 2.197 | -1.753 | -0.001201 |
| H28 | hc | -1.871 | 1.903 | -1.47 | -0.001201 |
| C18 | c3 | -1.295 | 4.533 | -0.727 | 0.017203 |
| H29 | hc | -1.693 | 3.029 | 0.774 | -0.008868 |
| H30 | hc | 0.014 | 3.321 | 0.493 | -0.008868 |
| C19 | c3 | -1.343 | 5.744 | 0.214 | 0.047346 |
| H31 | hc | -0.556 | 4.72 | -1.52 | -0.002687 |
| H32 | hc | -2.264 | 4.432 | -1.236 | -0.002687 |
| C20 | c3 | -1.678 | 7.055 | -0.503 | -0.077942 |
| H33 | hc | -2.083 | 5.558 | 1.004 | -0.006386 |
| H34 | hc | -0.375 | 5.843 | 0.724 | -0.006386 |
| H35 | hc | -1.707 | 7.898 | 0.195 | 0.014633 |
| H36 | hc | -0.934 | 7.287 | -1.273 | 0.014633 |
| H37 | hc | -2.655 | 6.998 | -0.997 | 0.014633 |
| C21 | c | 2.272 | -1.991 | -0.631 | 0.822622 |
| H38 | hc | 1.115 | -1.245 | 1.008 | 0.054487 |
| H39 | hc | 1.013 | -2.972 | 0.766 | 0.054487 |
| O5 | o | 2.285 | -2.027 | -1.844 | -0.570236 |

***Table S.3.*** Atom names, atom types, coordinates and charges for 3PF residue.

| **Atom name** | **Atom type** | **X** | **Y** | **Z** | **Charge** |
| --- | --- | --- | --- | --- | --- |
| O4 | os | 3.382 | -1.953 | 0.14 | -0.196577 |
| C22 | c3 | 4.672 | -1.972 | -0.544 | 0.092497 |
| C23 | c3 | 5.61 | -2.699 | 0.42 | -0.337276 |
| H40 | h1 | 4.56 | -2.559 | -1.458 | 0.126614 |
| C24 | c3 | 5.085 | -0.547 | -0.927 | -0.112599 |
| H41 | hc | 6.025 | -0.623 | -1.486 | 0.050422 |
| H42 | hc | 4.335 | -0.175 | -1.635 | 0.050422 |
| C25 | c3 | 5.233 | 0.442 | 0.237 | 0.070565 |
| C26 | c3 | 5.509 | 1.874 | -0.241 | 0.033821 |
| H43 | hc | 4.32 | 0.431 | 0.846 | -0.016721 |
| H44 | hc | 6.049 | 0.128 | 0.901 | -0.016721 |
| C27 | c3 | 5.682 | 2.879 | 0.905 | 0.001995 |
| H45 | hc | 6.412 | 1.88 | -0.869 | -0.006506 |
| H46 | hc | 4.687 | 2.202 | -0.892 | -0.006506 |
| C28 | c3 | 5.941 | 4.313 | 0.428 | 0.014559 |
| H47 | hc | 4.783 | 2.864 | 1.538 | -0.00775 |
| H48 | hc | 6.511 | 2.555 | 1.55 | -0.00775 |
| C29 | c3 | 6.119 | 5.318 | 1.573 | 0.041382 |
| H49 | hc | 6.838 | 4.328 | -0.209 | -0.002897 |
| H50 | hc | 5.11 | 4.638 | -0.214 | -0.002897 |
| C30 | c3 | 6.372 | 6.749 | 1.089 | -0.075888 |
| H51 | hc | 5.224 | 5.301 | 2.211 | -0.006678 |
| H52 | hc | 6.951 | 4.995 | 2.212 | -0.006678 |
| H53 | hc | 6.494 | 7.44 | 1.93 | 0.015491 |
| H54 | hc | 7.28 | 6.806 | 0.478 | 0.015491 |
| H55 | hc | 5.54 | 7.114 | 0.477 | 0.015491 |
| C31 | c | 6.95 | -3.037 | -0.2 | 0.909896 |
| H56 | hc | 5.773 | -2.127 | 1.339 | 0.087215 |
| H57 | hc | 5.144 | -3.641 | 0.733 | 0.087215 |
| O6 | os | 7.804 | -3.504 | 0.735 | -0.426935 |
| O7 | o | 7.235 | -2.932 | -1.375 | -0.615711 |
| C32 | c3 | 9.099 | -3.895 | 0.246 | -0.044985 |
| H58 | h1 | 9.649 | -4.245 | 1.119 | 0.092666 |
| H59 | h1 | 9.006 | -4.693 | -0.494 | 0.092666 |
| H60 | h1 | 9.609 | -3.045 | -0.214 | 0.092666 |

*S.2 Atomic charges for polylactic acid*

Charge derivation procedure led to the creation of three fragments, called PDI, PDL and PDF for the first residue of the chain, bulk monomers and terminal residue of the chain, respectively (Figure S.2).


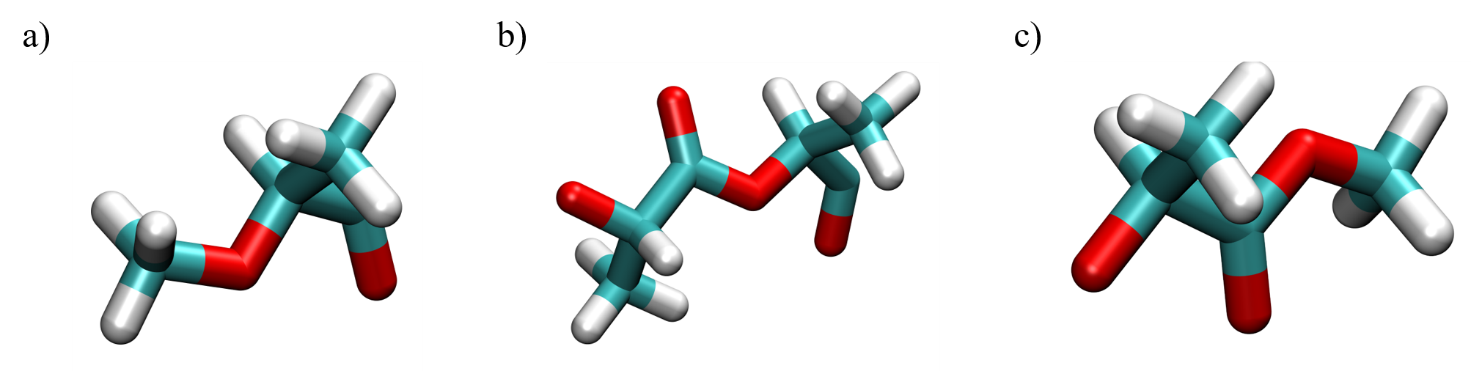


***Figure S.2.*** Building blocks for PDLA: PDI (a), PDL (b) and PDF (c).

Atom names, atom types (according to second generation GAFF force field), atomic charges and coordinates are reported in Table S.4 – S.6.

***Table S.4.*** Atom names, atom types, coordinates and charges for PDI residue.

| **Atom name** | **Atom type** | **X** | **Y** | **Z** | **Charge** |
| --- | --- | --- | --- | --- | --- |
| C1 | c3 | -5.793 | -0.209 | 0.407 | 0.226526 |
| O1 | os | -6.972 | -0.071 | -0.365 | -0.33054 |
| C2 | c | -4.629 | 0.234 | -0.474 | 0.331453 |
| H1 | h1 | -5.818 | 0.476 | 1.27 | 0.002998 |
| C3 | c3 | -5.579 | -1.646 | 0.907 | -0.170651 |
| C4 | c3 | -8.156 | 0.067 | 0.409 | -0.022922 |
| H2 | h1 | -8.97 | 0.251 | -0.294 | 0.068615 |
| H3 | h1 | -8.388 | -0.837 | 0.986 | 0.068615 |
| H4 | h1 | -8.085 | 0.917 | 1.103 | 0.068615 |
| H5 | hc | -4.64 | -1.727 | 1.46 | 0.058143 |
| H6 | hc | -6.395 | -1.942 | 1.57 | 0.058143 |
| H7 | hc | -5.551 | -2.336 | 0.059 | 0.058143 |
| O3 | o | -4.686 | 0.489 | -1.656 | -0.417137 |

***Table S.5.*** Atom names, atom types, coordinates and charges for PDL residue.

| **Atom name** | **Atom type** | **X** | **Y** | **Z** | **Charge** |
| --- | --- | --- | --- | --- | --- |
| O2 | os | -3.497 | 0.288 | 0.266 | -0.178331 |
| C5 | c3 | -2.285 | 0.668 | -0.408 | 0.061887 |
| C6 | c | -1.172 | -0.196 | 0.172 | 0.312871 |
| H8 | h1 | -2.382 | 0.437 | -1.473 | 0.137858 |
| C7 | c3 | -2.026 | 2.163 | -0.22 | -0.266165 |
| H9 | hc | -1.082 | 2.442 | -0.689 | 0.094513 |
| H10 | hc | -2.834 | 2.733 | -0.683 | 0.094513 |
| H11 | hc | -1.982 | 2.413 | 0.844 | 0.094513 |
| O4 | os | -0.015 | 0.098 | -0.455 | -0.178331 |
| O5 | o | -1.291 | -1.014 | 1.056 | -0.351658 |
| C8 | c3 | 1.169 | -0.603 | -0.025 | 0.061887 |
| C9 | c | 2.311 | 0.407 | -0.077 | 0.312871 |
| H12 | h1 | 1.034 | -0.922 | 1.012 | 0.137858 |
| C10 | c3 | 1.416 | -1.816 | -0.921 | -0.266165 |
| H13 | hc | 2.34 | -2.316 | -0.624 | 0.094513 |
| H14 | hc | 0.586 | -2.519 | -0.823 | 0.094513 |
| H15 | hc | 1.499 | -1.51 | -1.967 | 0.094513 |
| O7 | o | 2.228 | 1.556 | -0.447 | -0.351658 |

***Table S.6.*** Atom names, atom types, coordinates and charges for PDF residue.

| **Atom name** | **Atom type** | **X** | **Y** | **Z** | **Charge** |
| --- | --- | --- | --- | --- | --- |
| O6 | os | 3.44 | -0.186 | 0.359 | -0.226719 |
| C11 | c3 | 4.648 | 0.602 | 0.358 | 0.055763 |
| C12 | c | 5.774 | -0.337 | -0.063 | 0.58489 |
| H16 | h1 | 4.553 | 1.391 | -0.393 | 0.109176 |
| C13 | c3 | 4.873 | 1.216 | 1.738 | -0.164171 |
| H17 | hc | 5.804 | 1.787 | 1.744 | 0.069994 |
| H18 | hc | 4.047 | 1.889 | 1.98 | 0.069994 |
| H19 | hc | 4.93 | 0.437 | 2.503 | 0.069994 |
| O8 | os | 6.926 | 0.347 | -0.134 | -0.36265 |
| O9 | o | 5.66 | -1.519 | -0.304 | -0.448722 |
| C14 | c3 | 8.09 | -0.415 | -0.521 | -0.033385 |
| H20 | h1 | 8.917 | 0.294 | -0.521 | 0.091945 |
| H21 | h1 | 8.273 | -1.218 | 0.195 | 0.091945 |
| H22 | h1 | 7.95 | -0.84 | -1.517 | 0.091945 |

*S.3 Summary of performed simulations*

***Table S.7.*** Detailed summary of performed simulations. ^a^Box size of equilibrated system. ^b^Values for each window.

| **System** | **Number of atoms** | **Water molecules** | **Ions** | **Box size^a^**  **[nm x nm x nm]** | **Simulation time**  **[ns]** |
| --- | --- | --- | --- | --- | --- |
| PDLA in water | 7470 | 2469 | 0 | 5.40 x 3.82 x 3.68 | 50 |
| P3HD in water | 17613 | 5808 | 0 | 5.84 x 5.51 x 5.61 | 50 |
| DOPC | 31890 | 4736 | 9 Na+  9 Cl- | 6.80 x 6.80 x 6.58 | 150 |
| DOPC/PDLA^b^ | 47781 | 10000 | 27 Na+  27 Cl- | 6.82 x 6.82 x 10.05 | 80 |
| DOPC/P3HD^b^ | 47907 | 10000 | 27 Na+  27 Cl- | 6.81 x 6.81 x 10.11 | 80 |
| PDLA CG | 7 | 1556 | 0 | 5.71 x 5.71 x 5.71 | 160 |
| P3HD CG | 19 | 3202 | 0 | 7.26 x 7.26 x 7.26 | 160 |
| DOPC CG | 3683 | 2099 | 24 Na+  24 Cl- | 6.53 x 6.53 x 9.78 | 600 |
| DOPC/PDLA CG WTMD | 3689 | 2098 | 24 Na+  24 Cl- | 6.53 x 6.53 x 9.85 | 18000 |
| DOPC/P3HD CG WTMD | 3684 | 2081 | 24 Na+  24 Cl- | 6.63 x 6.63 x 9.57 | 22000 |

*S.4 Convergence of atomistic simulations*

80 ns molecular dynamics simulations were performed for each window. The first 50 ns were employed for equilibration and discarded; free energy was computed using the last 30 ns as an average of three blocks of 10 ns each. Results are shown in Figure S.3 and S.4 for PDLA and P3HD, respectively.


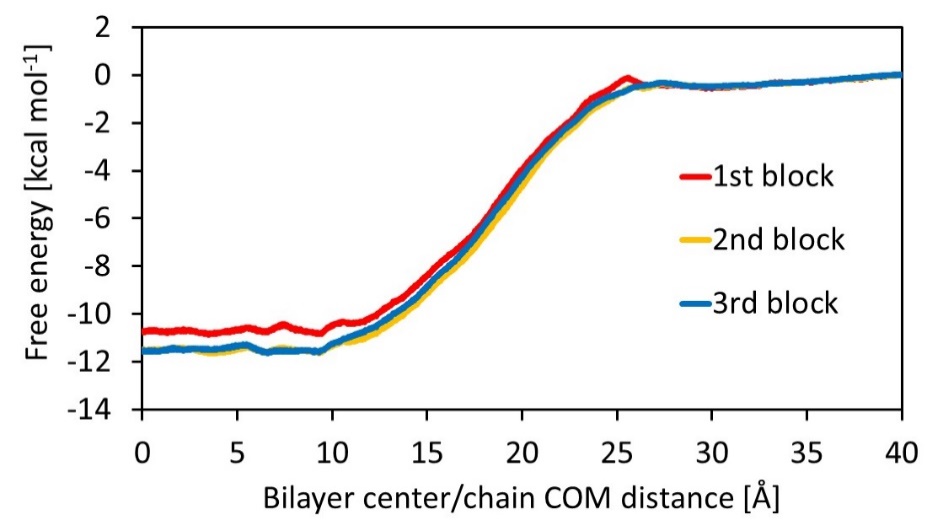


***Figure S.3.*** Free energy for PDLA/DOPC system as a function of simulation length.


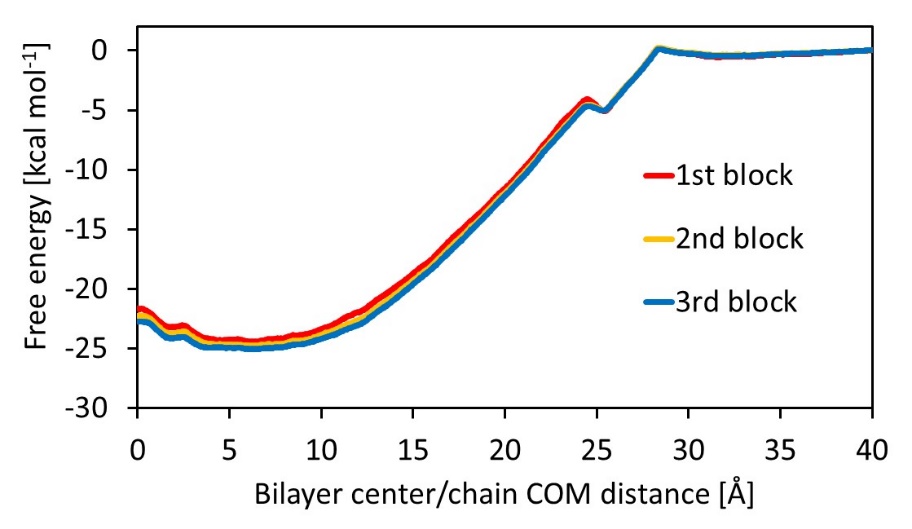


***Figure S.4.*** Free energy for P3HD/DOPC system as a function of simulation length.

*S.5 Data summary*

***Table S.8.*** Data obtained from inhomogeneous solubility – diffusion model.

|  | **PDLA** | **PHB** |
| --- | --- | --- |
| ***ΔG^0^_bind_* [kcal mol^-1^]** | -11.49 ± 0.69 | -23.85 ± 0.99 |
| ***K_lip_* [-]** | 2.20 ± 1.97· 10^8^ | 1.53 ± 1.30 · 10^17^ |
| ***P_eff_* [cm s^-1^]** | 1.83 ± 0.20· 10^-8^ | 1.31 ± 0.39 · 10^-8^ |


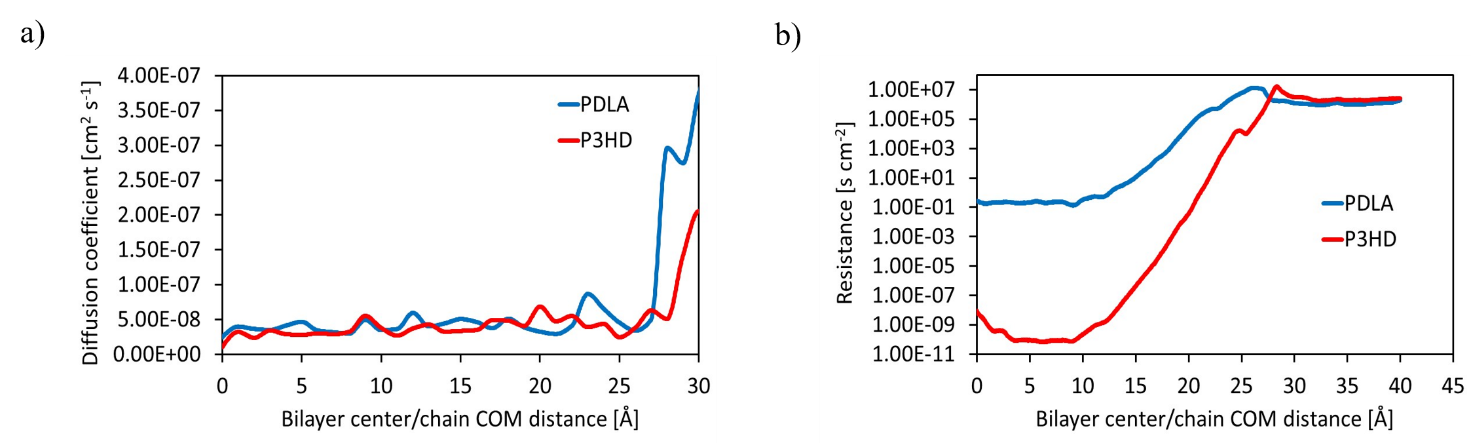


***Figure S.5.*** Position dependent diffusion coefficient (a) and resistance (b) for PDLA and P3HD.

*S.6 Histograms from Umbrella Sampling*


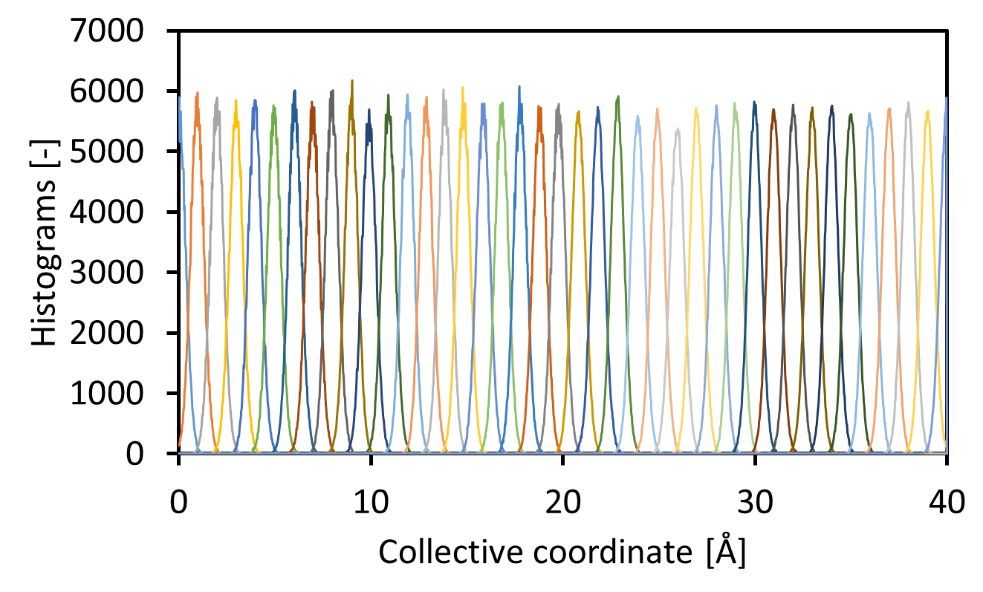


***Figure S.6.*** Histograms from Umbrella Sampling for PDLA.


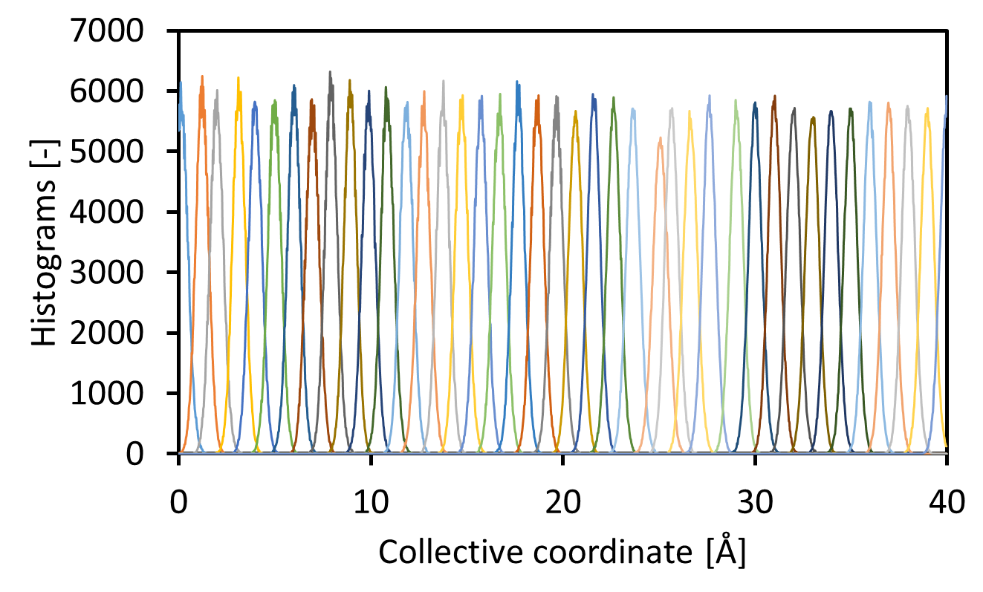


***Figure S.7.*** Histograms from Umbrella Sampling for PHB.

*S.7 Parameterization of bonded interactions for coarse-grained model*

Parameters for bonded interactions were estimated starting from MD simulations of the single oligomers in water solution, in order to best reproduce bond lengths, angle and dihedral distributions.

Potential energy contribution due to covalent bonds *V_b_* is described with a simple harmonic potential:

$V_{b}(r)=\frac{1}{2}k_{b}{(r-r_{0})}^{2}$ (S.1)

where k_b_ is the force constant, *r* is bond length and *r_0_* is the equilibrium bond length.

The contribution due to angle bending is described through the sum of two functions: a cosine-based harmonic potential *V_a_* and a restricted bending potential *V_rb_*, whose aim is to prevent that the angle reaches 180° value since this constitutes a known source of numerical instabilities in coarse-grained simulation.

$V_{a}\left( \theta\right)=\frac{1}{2}k_{\theta,1}\left[ \cos\left( \theta\right)-cos(\theta_{0}) \right]^{2}+\frac{1}{2}k_{\theta,2}\frac{\left[ \cos\left( \theta\right)-cos(\theta_{0}) \right]^{2}}{{sin}^{2}\theta}$ (S.2)

where *θ* is the angle value, *θ_0_* is the equilibrium angle value and *k_θ,1_* and *k_θ,2_* are force constants.

The contribution related to dihedral angles *V_d_* is accounted for through a sum of periodic functions:

$V_{d}\left( \varphi\right)=\sum_{n=1}^{N} k_{\varphi,n}\left[ 1+cos(n\varphi-\varphi_{s,n}) \right]$ (S.3)

where *φ* is the dihedral angle, *n* is multiplicity, *φ_s_* is reference dihedral angle and *k_φ,n_* are force constants.

Bond length, angle and dihedral distribution of the atomistic system were taken from the simulations of the single oligomers in water phase described in the manuscript. The analogous quantities for the coarse-grained simulations were obtained with 160 ns in NPT ensemble at 310 K and 1 atm.

Results are described in detail in the following paragraphs.

*S.7.1 PDLA*

Comparison between bond lengths, angle and dihedral distributions obtained from full atomistic and coarse-grained simulations is shown in Figure S.8. PDLA was modeled using only C5 beads, therefore only C5 – C5 bonds, C5 – C5 – C5 angles and C5 – C5 – C5 – C5 dihedrals are present.


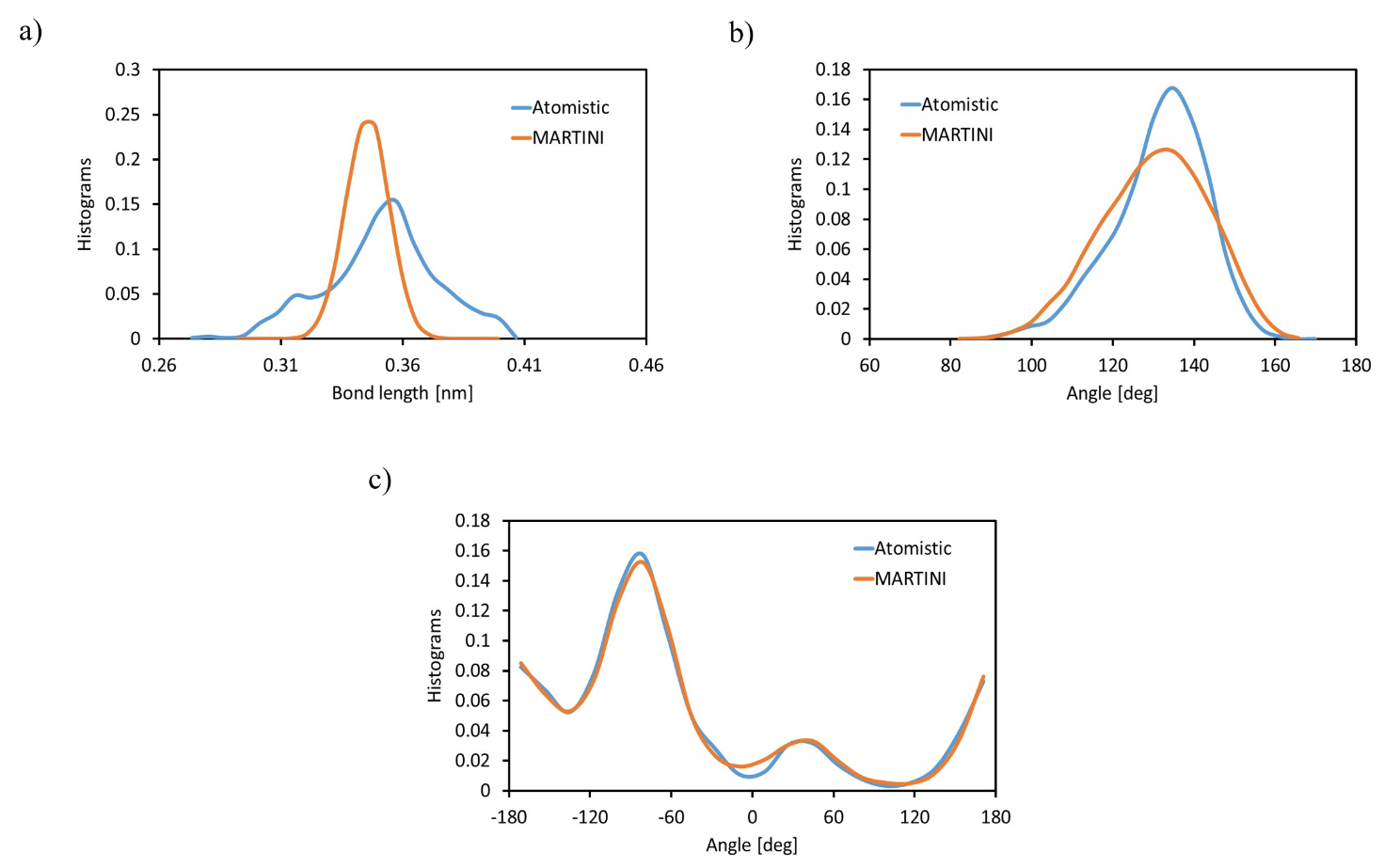


***Figure S.8.*** Comparison between bond length (a), angle (b) and dihedral angle (c) distributions in full atomistic and coarse-grained simulations. Distribution are averaged over all bonds, angles and dihedral angles.

Parameters are summarized in Table S.9; data are reported so that they can be easily implemented in a GROMACS topology file.

***Table S.9.*** Parameters for bonded interactions of PDLA. ^a^Potential energy function implemented in GROMACS. ^b^Force constant.

|  | **Type** | **funct^a^** | **k [kJ mol^-1^]^b^** | **r_0_ [nm]** |  |
| --- | --- | --- | --- | --- | --- |
| **Bond** | C5 – C5 | 1 | 4000 | 0.36 |  |
|  | **Type** | **funct** | **k [kJ mol^-1^]** | **θ_0_ [deg]** |  |
| **Angle** | C5 – C5 – C5 | 2 | 16.0 | 135.0 |  |
|  | C5 – C5 – C5 | 10 | 20.6 | 135.0 |  |
|  | **Type** | **funct** | **k [kJ mol^-1^]** | **φ_s_ [deg]** | **n** |
| **Dihedral** | C5 – C5 – C5 – C5 | 9 | 3.13 | 74.0 | 1 |
|  | C5 – C5 – C5 – C5 | 9 | -0.82 | 67.0 | 2 |
|  | C5 – C5 – C5 – C5 | 9 | 1.92 | -40.0 | 3 |

*S.7.2 P3HD*

The coarse-grained model of P3HD involves three different MARTINI beads: Na, C3 and C1.

This allows identifying three different kinds of bonds (Na – C3, Na – Na, C3 – C1), angles (Na – Na – Na, Na – C3 – C1, C3 – Na – Na) and dihedral angles (Na – Na – Na – Na, C1 – C3 – Na – Na, C3 – Na – Na – C3). Comparison between distributions obtained from full atomistic and coarse-grained simulations are shown in Figure S.9., Figure S.10 and Figure S.11 for bond lengths, angles and dihedral angles, respectively.


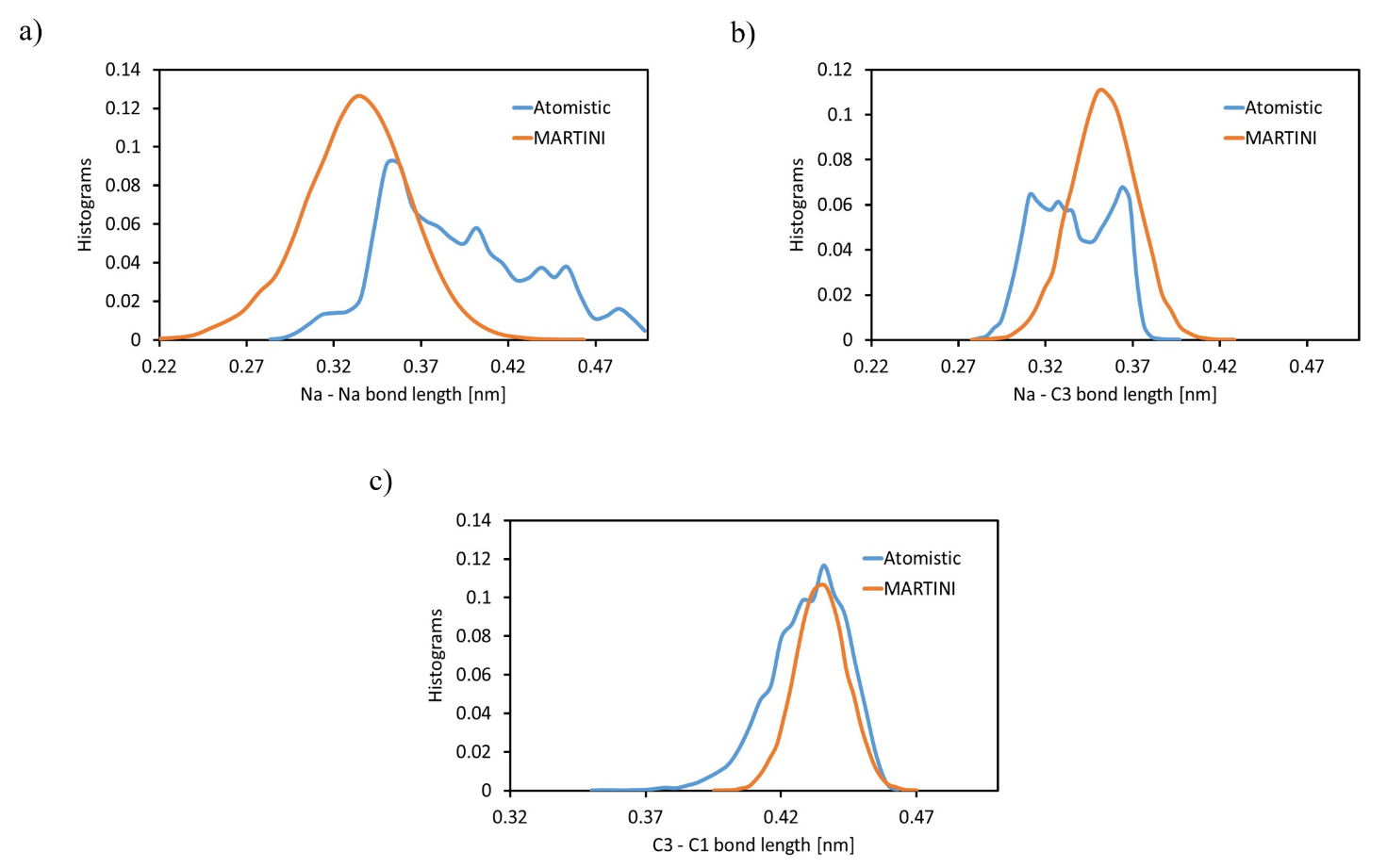


***Figure S.9.*** Comparison between Na – Na bond length (a), Na – C3 bond length (b) and C3 – C1 bond length (c) distributions in full atomistic and coarse-grained simulations. Distribution are averaged over all bonds.


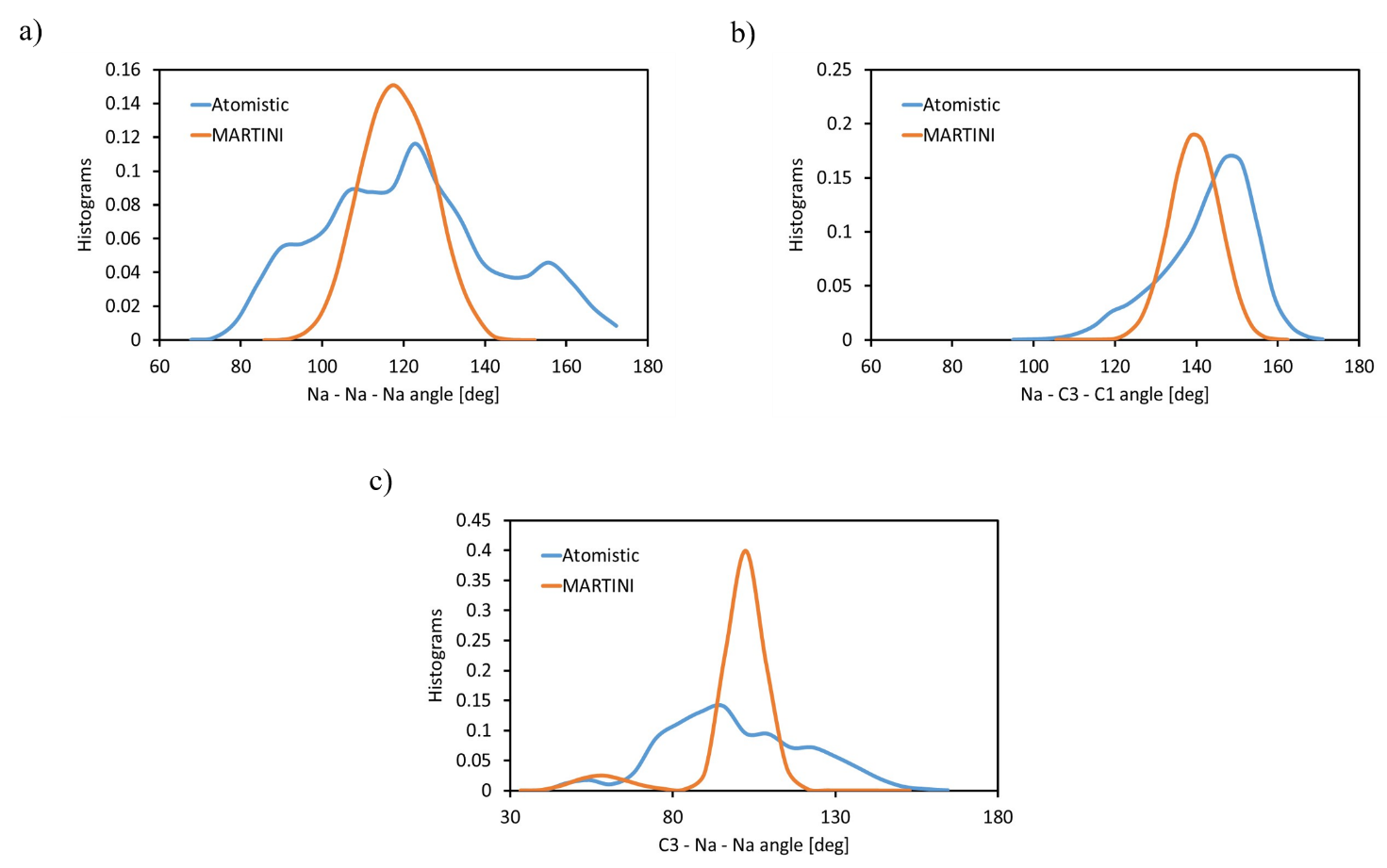


***Figure S.10.*** Comparison between Na – Na – Na angle (a), Na – C3– C1 angle (b) and C3 – Na – Na angle (c) distributions in full atomistic and coarse-grained simulations. Distribution are averaged over all angles.


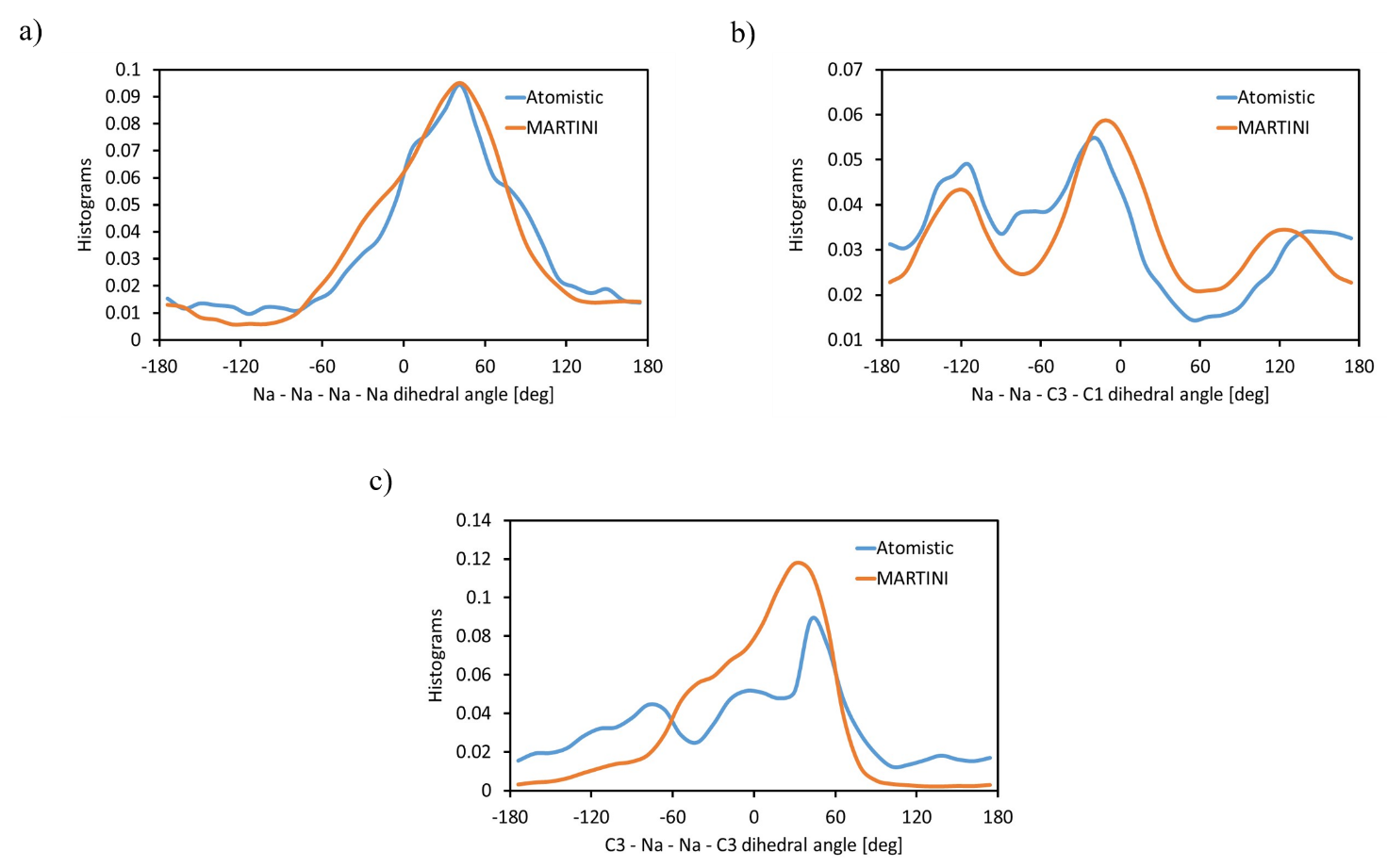


***Figure S.11.*** Comparison between Na – Na – Na – Na dihedral angle (a), Na – Na – C3 – C1 dihedral angle (b) and C3 – Na – Na – C3 dihedral angle (c) distributions in full atomistic and coarse-grained simulations. Distribution are averaged over all dihedral angles.

***Table S.10.*** Parameters for bonded interactions of P3HB. ^a^Potential energy function implemented in GROMACS. ^b^Force constant.

|  | **Type** | **funct^a^** | **k [kJ mol^-1^]^b^** | **r_0_ [nm]** |  |
| --- | --- | --- | --- | --- | --- |
| **Bond** | Na – Na | 1 | 1500 | 0.35 |  |
|  | Na – C3 | 1 | 1500 | 0.36 |  |
|  | C3 – C1 | 1 | 3000 | 0.44 |  |
|  | **Type** | **funct** | **k [kJ mol^-1^]** | **θ_0_ [deg]** |  |
| **Angle** | Na – Na – Na | 2 | 0.66 | 122.0 |  |
|  | Na – Na – Na | 10 | 14.91 | 122.0 |  |
|  | C1 – C3 – Na | 2 | 32.75 | 147.0 |  |
|  | C1 – C3 – Na | 10 | 15.05 | 147.0 |  |
|  | C3 – Na – Na | 2 | 0.47 | 95.5 |  |
|  | C3 – Na – Na | 10 | 15.41 | 95.5 |  |
|  | **Type** | **funct** | **k [kJ mol^-1^]** | **φ_s_ [deg]** | **n** |
| **Dihedral** | Na – Na – Na - Na | 9 | 2.96 | -138.0 | 1 |
|  | Na – Na – Na – Na | 9 | -0.28 | 58.5 | 2 |
|  | Na – Na – Na - Na | 9 | 0.49 | 51.6 | 3 |
|  | Na – Na – C3 – C1 | 9 | 1.08 | 84.8 | 1 |
|  | Na – Na – C3 – C1 | 9 | 0.67 | 132.7 | 2 |
|  | Na – Na – C3 – C1 | 9 | -0.50 | -8.5 | 3 |
|  | Na – Na – C3 – C1 | 9 | 0.19 | 66.6 | 4 |
|  | C3 – Na – Na – C3 | 9 | 1.47 | 169.0 | 1 |
|  | C3 – Na – Na – C3 | 9 | 0.72 | -90.0 | 2 |
|  | C3 – Na – Na – C3 | 9 | 0.51 | -67.0 | 3 |
|  | C3 – Na – Na – C3 | 9 | 0.20 | 60.5 | 4 |

*S.8 Convergence of Well-Tempered Metadynamics*


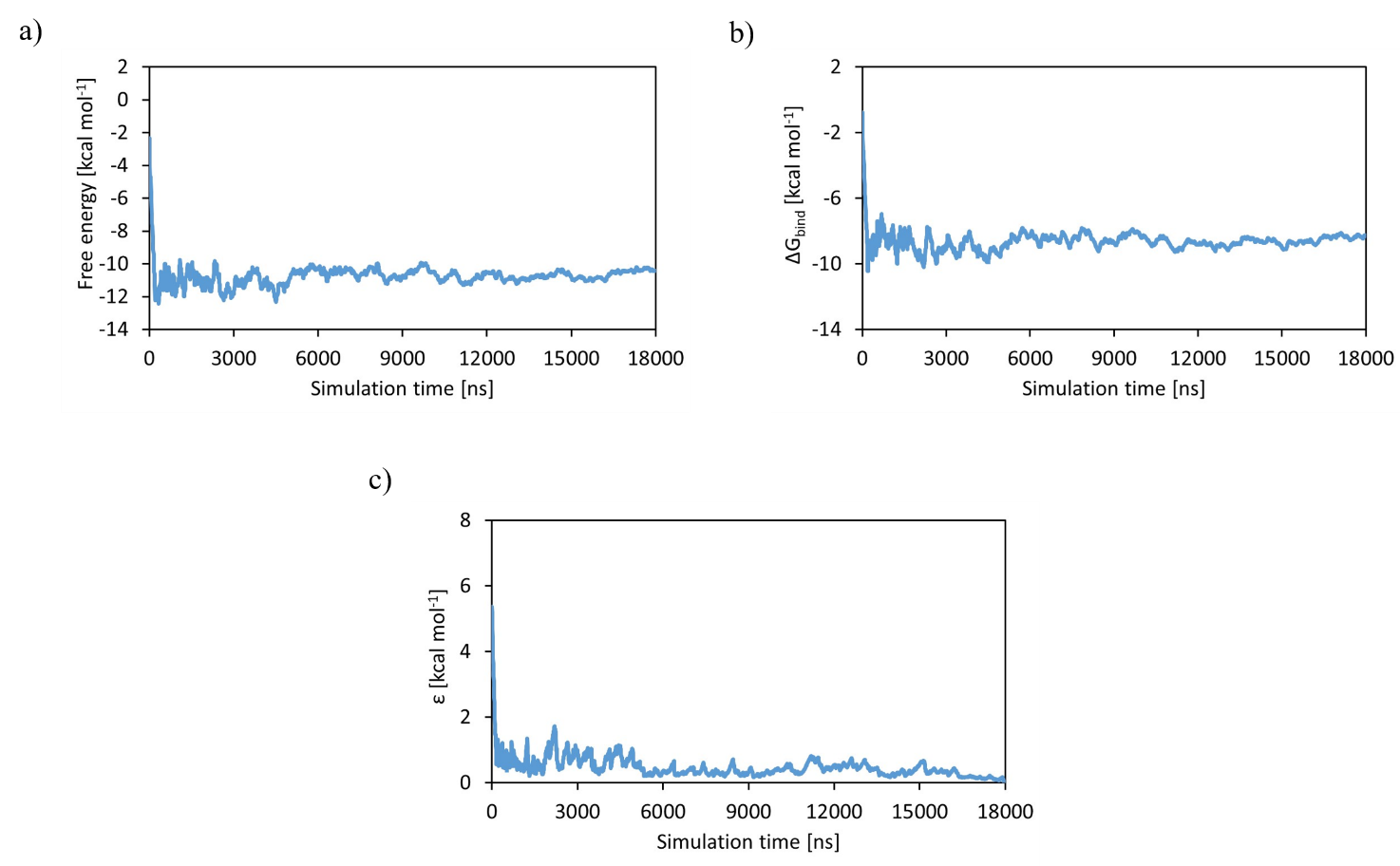


***Figure S.12.*** Difference in free energy between lipid and water phase (a), *ΔG*_bind_ (b) and error *ε* (computed through eq. 8 in the main text) (c) as a function of simulation time for PDLA oligomer.


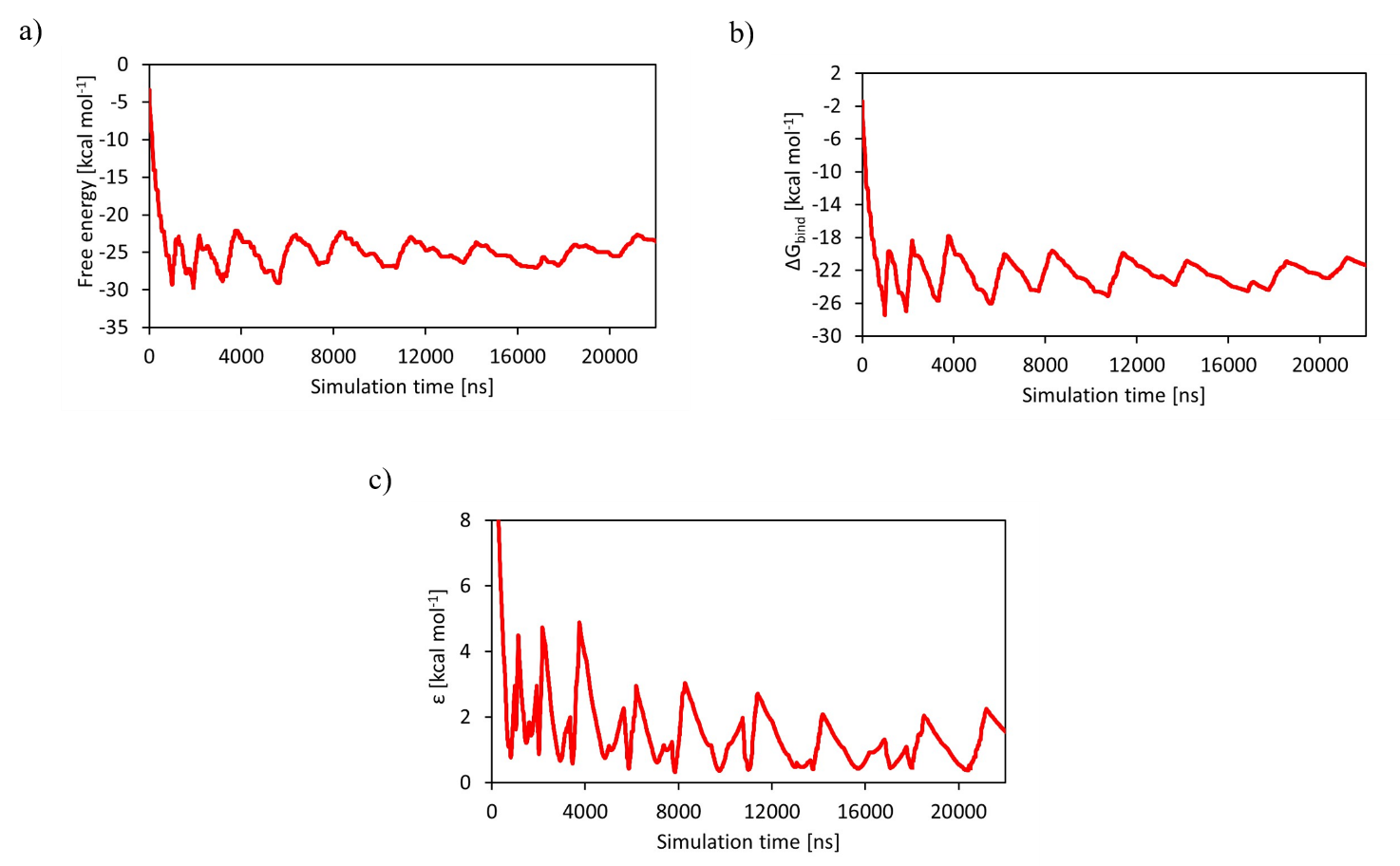


***Figure S.13.*** Difference in free energy between lipid and water phase (a), *ΔG*_bind_ (b) and error *ε* (computed through eq. 8 in the main text) (c) as a function of simulation time for P3HD oligomer.
